# Task-specific computational fluid dynamics evaluation of multi-outlet extrusion nozzles for bioprinting

**DOI:** 10.3389/fbioe.2026.1784513

**Published:** 2026-04-17

**Authors:** Cesar D. Vargas Urdaneta, Michael Taynnan Barros

**Affiliations:** School of Computer Science and Electronic Engineering, University of Essex, Colchester, United Kingdom

**Keywords:** 3D bioprinting, acellular, bioinks, biomaterials, bioprinting, branch angles, branching angle, CAD design

## Abstract

**Introduction:**

Extrusion bioprinting is transitioning from proof-of-concept demonstrations toward repeatable, manufacturing-style workflows, yet nozzle design remains a key bottleneck. Within compact, opaque channels, bioinks experience geometry-driven shear, pressure losses, and flow redistribution that directly affect cell viability, extrusion stability, and deposition uniformity. Multi-outlet nozzles offer increased throughput by splitting a single feed into parallel filaments, but often suffer from flow imbalance and junction-induced shear hotspots. As a result, nozzle selection is still largely guided by trial-and-error rather than quantitative design evidence.

**Methods:**

This study presents a controlled, task-oriented comparison of two multi-outlet splitter archetypes—a radial 90° manifold and a branched Y-split—each implemented with two and four outlets. Three-dimensional computational fluid dynamics (CFD) simulations were performed using representative rheological models for common hydrogel bioinks (GelMA, MeHA, and alginate) under pneumatic actuation. Internal pressure, velocity, and wall shear stress fields were resolved and translated into practical performance metrics, including outlet flow balance and pressure-normalised throughput.

**Results:**

The two-outlet 90° manifold consistently produced the most uniform flow distribution and lowest shear exposure across shear-thinning bioinks, establishing it as the most robust configuration for cell-laden and precision printing. The two-outlet Y-split achieved higher outlet velocities, supporting faster deposition, but introduced elevated shear at junctions and greater sensitivity to operating conditions. Increasing the outlet count to four significantly increased flow maldistribution across all geometries and conditions, while failing to eliminate junction-driven shear hotspots, particularly in Y-split designs.

**Discussion:**

These findings demonstrate that nozzle geometry is a primary control parameter in extrusion bioprinting and cannot be reliably scaled by symmetry alone. The results establish practical, evidence-based guidelines for selecting nozzle architectures based on application requirements, including cell safety, precision, and throughput. The proposed framework enables more predictable nozzle design, improves reproducibility, and defines safer operating windows for bioprinting with living cells.

## 1 Introduction

Three-dimensional (3D) bioprinting, the layer-by-layer deposition of biomaterials and living cells, has become an important technology for engineering tissues, organ models, and cell-laden constructs while providing precise control over the placement of cells and biomaterials at both millimetre and micro-scale levels (Murphy and Atala, 2014; Mandrycky et al., 2016). A diverse toolbox of printing methods now enables the controlled placement of biomaterials and living cells with steadily improving control over the positioning of biomaterials and cells (Yilmaz et al., 2021). These methods include pneumatic and piston-driven extrusion, inkjet and drop-on-demand deposition, light-based printing (digital light processing or DLP, two-photon, and volumetric approaches), and embedded or freeform printing (extrusion into yield-stress support baths) (Hölzl et al., 2016; Derby, 2012; Melchels et al., 2010; Hinton et al., 2015). The field is transitioning from proof-of-concepts to manufacturing-oriented workflows that balance print quality, viability, and repeatability against speed and cost (Sun et al., 2020; Ma et al., 2025).

**FIGURE 1:**
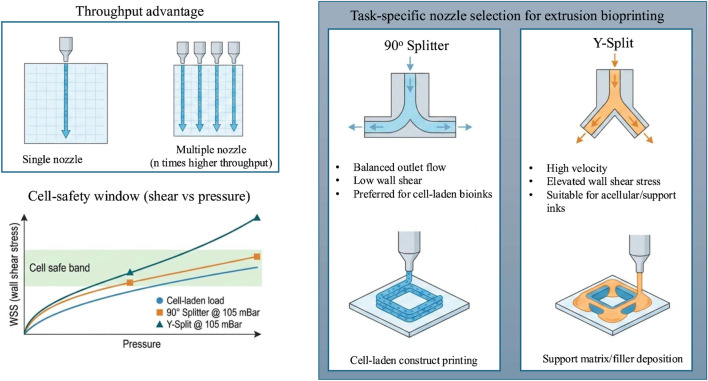
Overview of the extrusion bioprinting workflow, from CAD design to construct application. The process progresses through design, material preparation, printing, and post-print maturation before final assembly or implantation. This study focuses on the printing stage, where nozzle geometry dictates flow behaviour and cell stress.

Extrusion bioprinting begins with digital design and material formulation, followed by the controlled deposition of bioinks during the printing phase, and concludes with post-printing maturation and assembly. This study focuses specifically on the printing stage, where nozzle geometry governs how applied pressure is transformed into internal velocity fields and wall shear stresses, as illustrated in Figure 1. These fluid-dynamic factors ultimately determine flow balance, print fidelity, and the mechanical environment experienced by embedded cells. Despite this progress, several challenges limit routine, high-quality bioprinting. Print fidelity can degrade through non-uniform flow, strand swelling, and die swell (extrudate expansion at the nozzle exit) (Hölzl et al., 2016). Cell viability can be reduced by excessive wall shear stress (WSS, the frictional force per unit area exerted by flow on channel walls) and extensional stresses (Murphy and Atala, 2014; Mandrycky et al., 2016; Li et al., 2025). Practical operation must also respect pressure limits, footprint constraints, and requirements for sterilisable hardware (Sun et al., 2020). Many of these issues originate inside the nozzle, where complex, non-Newtonian bioinks experience geometry-dependent pressure drops and shear fields (Reina-Romo et al., 2021; Gómez-Blanco et al., 2023; Gharraei et al., 2024). However, computational fluid dynamics (CFD) studies that test different nozzle designs and operating conditions are still limited, so most optimisation relies on trial-and-error and leaves little basis for making informed design decisions (Gómez-Blanco et al., 2023; Zhou et al., 2024).

Nozzle geometry is a major design parameter that influences cell-compatible flow conditions and process performance through factors such as branching angle, curvature, taper, and outlet count (Blanco et al., 2024; Malekpour and Chen, 2022). Because in-nozzle phenomena (pressure, velocity, and shear-stress fields) are difficult to measure experimentally, CFD is a practical tool to evaluate potential nozzle designs before prototyping (Fareez et al., 2024; Blanco et al., 2024; Ates and Bartolo, 2023). With appropriate rheological models for shear-thinning bioinks and calibrated boundary conditions, simulations can map pressure, velocity, and WSS fields, quantify outlet flow balance (uniformity of discharge rates across multiple nozzles), and predict safe operating windows that are otherwise costly to explore experimentally (Müller et al., 2023; Guo et al., 2023).

Designing extrusion nozzles requires balancing competing requirements that depend on the intended printing task. Geometric choices (branch angles, curvature, taper, outlet count, and path-length symmetry), operating conditions (pressure and flow control), bioink rheology, and printer integration constraints (sterilisability and footprint) interact in non-trivial ways to determine in-nozzle flow behaviour and, ultimately, printing performance (Müller et al., 2023). Within this design space, multi-outlet architectures are attractive because they can increase throughput by splitting a single inlet into multiple, parallel deposition streams. However, increasing outlet number can also amplify outlet-to-outlet variability and flow imbalance (Fareez et al., 2024; Blanco et al., 2024). Likewise, geometric smoothing intended to reduce hydraulic losses can redistribute stresses and create elevated shear hotspots near junctions (Guo et al., 2023). These trade-offs make it unlikely that a single “best” nozzle geometry exists across applications. Instead, nozzle design should be *task-specific*, prioritising different objectives for cell-laden printing (minimising wall shear stress and maintaining uniform outlet flow) *versus* acellular, high-throughput deposition (maximising speed under acceptable shear and pressure limits). Multi-outlet nozzles therefore require a principled framework to determine when added outlets improve performance for a given task, and how the internal branching should be configured to control balance, shear exposure, and pressure drop. CFD provides this framework by resolving otherwise inaccessible in-nozzle fields (pressure, velocity, and wall shear) for realistic non-Newtonian inks and enabling controlled parameter sweeps over geometry, rheology, and operating pressure. Importantly, CFD quantifies task-relevant trade-offs (flow balance, shear hotspots, and pressure losses) with reproducible metrics before fabrication, allowing unsuitable designs to be eliminated early and safe operating windows for cell-laden extrusion to be bounded. This simulation-first approach focuses manufacturing and bench testing on a small set of evidence-supported prototypes, reducing time, material use, and cost while improving the likelihood that the final nozzle meets task-specific performance targets.

We therefore propose and study *task-specific multi-outlet nozzle* designs combining two elements: (i) a comparison of two archetypal multi-outlet geometries—a radial 90 ° splitter and a branched Y-splitter—across outlet counts; and (ii) evaluation against task-specific objectives that reflect practical priorities (high-throughput supports, precision patterning, and cell-safety-constrained extrusion). We focus on the mapping of geometry to application through quantitative metrics, thus moving beyond one-size-fits-all optimization. We build a reproducible CFD pipeline of 2- and 4-outlet nozzles with shear-thinning, power-law rheology representative of common bioinks (e.g., 10% gelatin methacrylate or GelMA, 2% methacrylated hyaluronic acid or MeHA, and 8% alginate) under pneumatic actuation (65 mbar–105 mbar) (Sharma et al., 2020). We compute spatial fields (static pressure, velocity magnitude, and WSS) and derive scalar metrics that capture performance: outlet flow imbalance, total pressure drop, and pressure-normalised throughput, defined as the mean outlet velocity divided by the inlet–outlet pressure drop (Chand et al., 2022). We report field maps, outlet symmetry, efficiency comparisons, and multi-metric trade-offs.

This work makes four specific contributions.

1. A *task-specific* evaluation framework for multi-outlet nozzles that formalises objectives for throughput, precision, and cell safety.
2. A comparative CFD study of two archetypes (90 ° radial and Y-split) at two outlet counts (Mandrycky et al., 2016; Hölzl et al., 2016), showing how outlet number interacts with geometry to shape pressure loss, velocity, WSS, and flow balance.
3. Introduction and use of practical, decision-making metrics (outlet flow imbalance and pressure-normalised throughput, $\eta ={\bar{U}}_{\text{out}}/\Delta p$ ).
4. Design guidance for practitioners: 90 ° 2-outlet nozzles as safe, balanced defaults for cell-laden printing; Y-split 2-outlet for fast, acellular deposition; and cautions for 4-outlet operation unless active balancing or control is available.

## 2 Methods

### 2.1 Methodology overview

**FIGURE 2:**
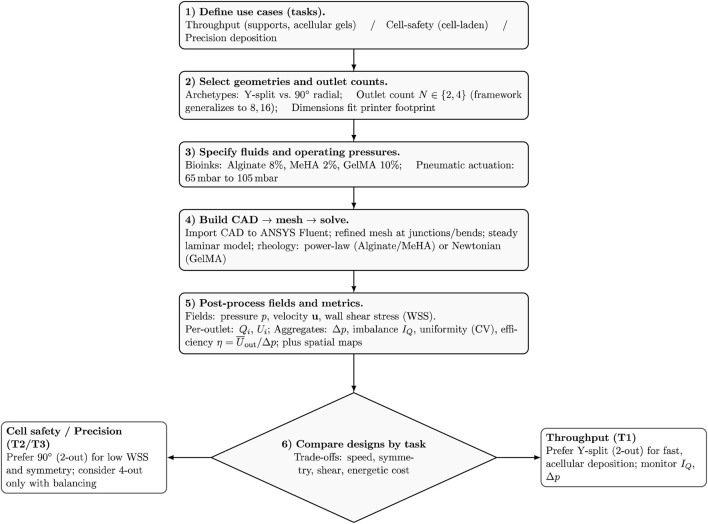
CFD-based workflow for task-specific evaluation of multi-outlet nozzles. Steps 1–5 define scenarios, solve the steady laminar problem with appropriate rheology, and extract comparable metrics. Step 6 maps results to task-oriented recommendations.

We assessed multi–outlet nozzle performance using a reproducible, simulation–driven workflow. The analysis considers three task classes: throughput-oriented printing (supports, acellular gels), cell-safety-constrained printing (cell-laden hydrogels), and precision deposition. We evaluate two archetypal geometries—a Y-split and a 90 ° radial splitter—at outlet counts of N=2 and N=4 , using dimensions selected to match the constraints of the target bioprinter. Three representative bioinks (8% alginate, 2% MeHA, 10% GelMA) under 65 mbar, 85 mbar and 105 mbar pneumatic actuation. CAD geometries are imported into ANSYS Fluent, locally refined meshes are generated at junctions and bends, and steady laminar simulations are run using appropriate rheological models (power-law or Newtonian). From each simulation we extract outlet flow rates, mean outlet velocities, mean and peak WSS, the inlet–outlet pressure drop, and pressure-normalised throughput $\left(\eta ={\bar{U}}_{\text{out}}/\Delta p\right)$ . These scalar metrics are complemented by spatial field maps of pressure, velocity, and WSS. Finally, a qualitative assessment of the trade–offs of the resulting (speed, symmetry, shear, energetic cost) recommends task–specific geometries. Figure 2 gives the general overview of our methodological computational framework, which is detailed in the following.

#### 2.1.1 Variables and scenario definition

**FIGURE 3:**
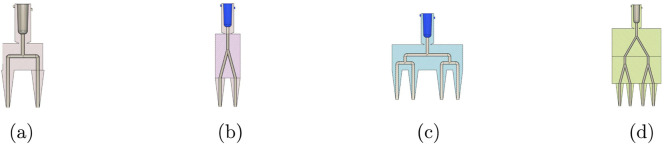
CAD renderings of the four multi-outlet nozzle configurations evaluated in this study: **(a)** 2-outlet 90 ° split, **(b)** 2-outlet Y-split, **(c)** 4-outlet 90 ° split, and **(d)** 4-outlet Y-split. All geometries share the same inlet and outlet diameters and were exported from the same STEP models used for meshing and CFD.

To keep the study focused and interpretable, variables were grouped into *design*, *fluid*, and *operating* sets. Table 1 summarizes the parameter space explored in this study and makes explicit what was varied *versus* held fixed to enable fair comparisons across nozzle designs. On the design side, we varied only the outlet count ( N=2 or 4) and the branching archetype (Y–split *versus* 90 ° radial), as shown in Figure 3, while keeping interface-critical dimensions (outlet diameter, spacing, and needle length) constant to match the target printer hardware. Geometric details such as curvature and bifurcation angles were set by the chosen archetype, and the overall footprint was constrained by practical integration and sterilisation requirements. On the fluid and operating sides, we varied the bioink formulation and inlet pressure (65 mbar–105 mbar gauge) while fixing outlet boundary conditions (0 mbar gauge), wall no-slip, and isothermal operation. This controlled scope isolates the influence of branching geometry and outlet count on flow balance and shear exposure. Two inks (8% alginate and 2% MeHA) were modeled as shear-thinning power-law fluids with representative (K,n) parameters, whereas 10% GelMA was approximated as Newtonian with a constant viscosity ( μ≈ 0.46Pa. s) under the simulated conditions. Density values were assigned per ink based on literature-informed assumptions to reflect modest formulation-dependent differences in inertia. These scenarios enable comparison of geometry effects across rheological regimes. We sampled 65 mbar, 85 mbar and 105 mbar to cover gentle cell-sparing operation (lower bound), typical lab settings (mid), and faster deposition regimes (upper bound), while remaining within the printer’s pressure capability and consistent with reported low-pressure extrusion ranges (50 mbar–100 mbar) (Sharma et al., 2020). Holding geometry and fluid properties fixed while varying pressure isolates each design’s hydraulic response, conversion of pressure into throughput, the resulting WSS, and sensitivity of outlet balance. This variation mirrors practical trade–offs between preserving cell viability (lower shear exposure) and increasing fabrication speed, and is consistent with CFD analyses showing that WSS increases with pressure under power-law rheology (Chand et al., 2022; Li et al., 2025).

**TABLE 1:** Study variables and scope. “Varied” indicates factors explored in this work; others were held fixed within practical constraints of the target printer and manufacturing route.

Category	Variable	Scope in this study
Design	Outlet count N	Varied: N=2,4 (framework generalizes to 8,16)
	Branching geometry	Varied: Y–split vs. 90 ° radial
	Outlet diameter, spacing, needle length	Fixed to fit printer hardware; consistent across cases
	Curvature/taper, bifurcation angle	Set per archetype; smooth transitions for 90 ° design
	Body thickness/footprint	Constrained by printer carriage and sterilisable envelope
Fluid	Bioink type	Varied: 8% alginate, 2% MeHA (power–law), 10% GelMA (Newtonian)
	Density	Fixed per ink
	Rheology parameters	Fixed per ink (power–law K,n or constant μ )
Operating	Inlet pressure	Varied: 65 mbar, 85 mbar and 105 mbar (gauge)
	Outlet condition	Fixed: 0 mbar (gauge), no–slip walls, isothermal

### 2.2 Evaluation framework

Each simulation corresponds to a single nozzle configuration defined by its geometry, bioink, and inlet pressure. The governing definitions for rheology, outlet flow, and performance metrics are given in Equations 1–9. Geometry is varied only in outlet count ( N=2 or 4) and branching layout (Y–split or 90 ° split); all other dimensions are kept identical across designs. Operating conditions are set by the imposed inlet pressure and the assigned bioink rheology.

For each configuration, steady-state CFD simulations are used to compute internal velocity, pressure, and wall shear stress fields. From these fields, outlet flow rates, pressure drop, and mean and peak wall shear stress are extracted and used to compare nozzle behaviour.

Bioink rheology: The rheological behaviour of shear-thinning bioinks was modelled using a power-law (Ostwald–de Waele) formulation:

$$\mu \left(\dot{\gamma }\right)=K\;{\dot{\gamma }}^{\;n-1},$$ (1)

where K is the consistency index and n ( <1 for shear–thinning materials) controls how strongly viscosity falls with shear rate. Each bioink has its own parameters (K,n) —for instance, 10% GelMA is nearly Newtonian, whereas 8% alginate and 2% MeHA are strongly shear–thinning. For a given configuration x , the CFD solver computes velocity u(r) , pressure p(r) , shear rate $\dot{\gamma }\left(r\right)$ , and wall shear stress $\tau_{w}\left(r\right)=\mu \left(\dot{\gamma }\right)\;{\dot{\gamma }}_{\|}$ along the nozzle walls.

#### 2.2.1 Metrics per–outlet

In fluid mechanics, quantities such as velocity are not uniform across a surface and vary spatially due to boundary effects. For example, flow is typically faster near the centre of an outlet and slower near the walls due to viscous drag. To interpret the CFD results in physically meaningful terms, outlet-level metrics were derived from surface-integrated quantities computed directly from the velocity field.

The primary outlet metric is the *volumetric flow rate* $Q_{i}$ , which determines the printing throughput, i.e., the amount of material discharged per unit time from outlet i∈O . It was defined as

$$Q_{i}\left(x\right)=\int_{A_{i}}u\cdot n\;dA,$$ (2)

where u is the velocity field, n is the unit normal vector to the outlet cross-section, and $A_{i}$ is the outlet area. This definition corresponds directly to the surface flux computed by the CFD solver.

A corresponding *mean outlet velocity* was derived consistently from the volumetric flow rate as

$${\bar{U}}_{i}\left(x\right)=\frac{Q_{i}\left(x\right)}{A_{i}},$$ (3)

where ${\bar{U}}_{i}$ is the area-averaged normal velocity at outlet i . Because all outlet areas are identical, imbalance computed from $Q_{i}$ is equivalent to imbalance computed from ${\bar{U}}_{i}$ .

Wall shear stress was not treated as a per-outlet metric. Instead, mean and peak wall shear stress were evaluated at the nozzle level as area-based quantities over the internal wetted wall surface, as defined in Section 2.2.2.

#### 2.2.2 Aggregate performance metrics

While per-outlet metrics reveal local behaviour, nozzle design decisions require system-level performance indicators that describe flow balance, hydraulic cost, and shear exposure within the nozzle.

To quantify how evenly flow is distributed across all outlets, the flow uniformity metric $\phi_{Q}\left(x\right)$ was defined as

$$\phi_{Q}\left(x\right)=\frac{1}{N}\overset{N}{\underset{i=1}{\sum }}{\left(\frac{Q_{i}-\bar{Q}}{\bar{Q}}\right)}^{\;2},$$ (4)

where $Q_{i}$ is the volumetric flow rate at outlet i , $\bar{Q}=\frac{1}{N}\sum_{i=1}^{N}Q_{i}$ is the mean outlet flow rate, and N is the total number of outlets. This metric represents the normalised variance of outlet flow rates and provides a global measure of flow balance, with values approaching zero indicating near-uniform splitting.

Because averaged measures can mask severe deviations at individual outlets, a complementary worst-case metric was also defined. The flow imbalance $I_{Q}\left(x\right)$ was computed as

$$I_{Q}\left(x\right)=\underset{i\in O}{\max}\frac{\left|Q_{i}-\bar{Q}\right|}{\bar{Q}},$$ (5)

where O={1,…,N} denotes the set of outlets. This metric identifies the most over- or under-fed outlet relative to the mean flow rate.

The hydraulic cost of each design was characterised using the pressure drop

$$\Delta p=\langle {p\rangle }_{\text{in}}-\langle {p\rangle }_{\text{out}},$$ (6)

where ⟨⋅⟩ denotes area-weighted averaging over the inlet and outlet planes. All pressures are reported as gauge pressures, with the outlet boundary prescribed at zero gauge pressure. Local regions of negative *gauge* pressure may appear in contour plots due to the chosen reference level (zero gauge at the outlets) and local acceleration effects. Design comparisons therefore use the pressure-drop metric Δp , rather than interpreting local gauge minima as an outlet condition.

In addition to flow and pressure metrics, shear exposure along the nozzle walls was quantified to characterise the mechanical environment experienced by the bioink. The mean wall shear stress was defined as an area-weighted surface average over the internal wetted wall region,

$${\bar{\tau }}_{w}\left(x\right)=\frac{\int_{\Gamma_{W}}\|\tau_{w}\|dA}{\int_{\Gamma_{W}}dA},$$ (7)

where $\left\|\tau_{w}\right\|$ is the wall shear stress magnitude and $\Gamma_{W}$ denotes the internal wall surface of the splitting block. The resulting value summarises the typical shear level within the nozzle.

Because localised shear peaks may occur even when the mean remains moderate, the peak wall shear stress was also evaluated as

$$\tau_{w,\max}\left(x\right)=\underset{\Gamma_{W}}{\max}\|\tau_{w}\|,$$ (8)

which identifies the maximum shear stress magnitude occurring anywhere on the same internal wall region $\Gamma_{W}$ and captures worst-case shear exposure.

Finally, pressure-normalised throughput was assessed using the efficiency index

$$\eta \left(x\right)=\frac{{\bar{U}}_{\text{out}}\left(x\right)}{\Delta p\left(x\right)}.$$ (9)

where ${\bar{U}}_{\text{out}}\left(x\right)$ is the mean outlet velocity averaged across all outlets and Δp(x) is the inlet–outlet pressure drop. This metric provides a compact measure of how effectively applied pressure is converted into outlet throughput.

#### 2.2.3 Task–specific performance criteria

Different bioprinting tasks place emphasis on different aspects of nozzle performance. High–throughput applications favour higher outlet velocities to increase deposition rate, whereas cell–laden printing prioritises reduced wall shear stress to limit shear–induced damage. Precision deposition, by contrast, requires highly uniform outlet flow together with stable, pressure–driven operation.

Rather than combining these competing requirements into a formal optimisation problem, nozzle designs were evaluated against task–specific performance criteria using directly computed CFD metrics. A throughput–oriented assessment focused on mean outlet velocity, interpreted alongside associated increases in wall shear stress and outlet flow imbalance. A cell–safety assessment emphasised both mean and peak wall shear stress as indicators of typical and worst–case shear exposure, with secondary consideration of flow uniformity. Precision–oriented assessment prioritised low outlet imbalance and moderate pressure drop while maintaining sufficient outlet velocity for stable extrusion.

Because practical printing conditions vary across bioink formulations and applied pressures, all designs were assessed consistently across the full set of simulated operating cases. Performance trends were therefore interpreted based on how robustly each geometry maintained acceptable velocity, shear, and flow balance across materials and pressure levels, rather than on behaviour under a single nominal condition.

To ensure biological and mechanical relevance, interpretation of the CFD results was guided by established feasibility limits reported in the literature. In particular, experimental studies have shown that high cell viability during extrusion is associated with limiting local wall shear stress and reducing the duration of cell exposure to high–shear regions (Blaeser et al., 2016). Similarly, practical printer constraints—including maximum allowable inlet pressure, manufacturable channel dimensions, and acceptable outlet flow imbalance as outlet count increases—were used to contextualise the results and identify designs likely to remain viable when scaled.

Together, these task–specific criteria define a realistic evaluation framework for comparing nozzle geometries. The analysis links CFD–resolved velocity, pressure, and wall shear stress fields directly to practical printing objectives without introducing a formal optimisation model or weighted objective function.

### 2.3 Designs

The nozzle geometry governs how flow divides and how shear develops during extrusion. To isolate geometric effects, four configurations were studied: two–outlet Y–split, two–outlet 90 ° split, four–outlet Y–split, and four–outlet 90 ° split. All models shared a common inlet channel (*inlet length* $L_{\text{in}}=8.50$ mm) and identical outlet diameters (*outlet diameter* $D_{\text{out}}=1.35$ mm) elected to match a commonly used extrusion scale for hydrogel bioprinting. Channel dimensions were chosen to match the resolution limits of the 3D-printed moulds (printable channels ≳ 1.2 mm, with walls thick enough to avoid leakage under pneumatic loading) and to remain within the bioprinter’s pressure limit (≤200 mbar) . Complete geometry specifications for all nozzle designs are reported in Supplementary Tables 7-10.

To ensure reproducibility while enabling meaningful comparison, each geometry was defined by a small set of *template parameters*.

- Outlet count N∈{2,4} .
- Branching archetype: Y–split (acute bifurcation) vs. orthogonal (90 ° ) split.
- Junction geometry: For Y–split designs, the bifurcation angles $\theta_{\text{Y}}$ and all other branching parameters are reported in the corresponding geometry tables. For 90 ° split designs, flow redirection was implemented using tangent–arc bends with a single, fixed bend radius $R_{c}$ defined per configuration.
- Curvature control: The internal bend radius $R_{c}$ and the straight sections before and after each turn were sized to minimise recirculation.
- Transition shaping: No taper within the split-block ( α = 0); tapering is applied only in the shared downstream needle geometry.

Dimensions were set to remain within the printhead’s available space and to preserve left-right symmetry, so that the flow paths on both sides had the same length and hydraulic resistance. Table 2 lists only geometry parameters common to all designs. All branching-specific dimensions are reported separately.

**TABLE 2:** Geometric parameters shared by all nozzle configurations. Exact split-block geometries are specified per nozzle in Supplementary Tables 6–9.

Item	Symbol/Value	Rationale
Inlet straight length	$L_{\text{in}}=8.50$ mm	Provides a stable, fully developed inlet flow before the first junction
Channel diameter (split-block)	D=1.35 mm	Constant diameter throughout the split-block to isolate geometric effects and ensure fair comparison across designs
Outlet diameter (at split-block exit)	$D_{\text{out}}=1.35$ mm	Fixed outlet size used across all configurations; shared needle geometry is defined separately (Supplementary Table 10)
Minimum printable diameter	≥1.2 mm	Ensures manufacturability and reduces clogging risk for hydrogel extrusion
Outlet count	N=2 or 4	Baseline and first scalable multi-outlet configurations studied
Internal taper (split-block)	α=0	No taper within the split-block; all channels maintain constant diameter. Tapering is applied only in the downstream needle section
Build planarity	Single build plane	Simplifies fabrication, cleaning, and meshing while preserving symmetry

#### 2.3.1 Two–outlet designs

The two–outlet geometries use a single bifurcation from the inlet to two identical outlets and serve as the baseline for assessing geometric effects. Each layout is mirror-symmetric, with the same path length and cross-section on both sides, so the ideal outcome is a 50:50 flow split. *Two–outlet Y–split:* The junction uses an acute bifurcation $\left(\theta_{\text{Y}}\right)$ to turn the flow gradually and reduce junction losses. Short tangent sections were added after the split to let the flow stabilise before entering the final taper and outlet. This design prioritises smoother streamlines and higher throughput at a given pressure, but requires a longer overall length. *Two–outlet 90* ° *split:* Flow is redirected into perpendicular branches via a single–radius fillet $\left(R_{c}\right)$ rather than a sharp right angle. The orthogonal layout is more compact and straightforward to fabricate and scale. The tighter turning can raise local WSS, but the symmetry and short path lengths favour balanced outlet flows and moderate pressure drops.

#### 2.3.2 Four–outlet designs

The four–outlet variants add a second, symmetric split to each branch (inlet → 2 → 4). The second junction duplicates the first (same design and shaping) to preserve path–length equality from inlet to all outlet. This layout tests the first stage of scaling: whether any small imbalance or high-shear region created at the first split becomes larger or smaller after the second split. *Four–outlet Y–split:* The four-outlet Y-split uses a two-stage angle strategy: a wider primary split followed by a narrower secondary split to keep flow redirection gentle at both junctions. This approach produces a smoother, more gradual branching layout, though at the cost of a longer overall path. *Four–outlet 90* ° *split:* In the four–outlet 90 ° split, each branch undergoes a second orthogonal split using the same bend radius $R_{c}$ and tangent lengths as the first tier, preserving geometric consistency across both stages.

### 2.4 Bioink models

#### 2.4.1 Density

Densities representative of water-based hydrogels were assigned as 1,050 kg m^−3^ for 8% alginate, 1,000 kg m^−3^ for 2% MeHA, and 1,030 kg m^−3^ for 10% GelMA (Jia et al., 2014; Petta et al., 2020; Pottathara et al., 2022). Density enters the inertial term of the momentum equation and is used for Reynolds number evaluation. In these low-Re, viscosity-dominated nozzle flows, it does not materially affect pressure drop or wall shear stress compared with rheology.

#### 2.4.2 Shear-thinning bioinks (alginate, MeHA)

The constitutive relations used to model bioink rheology are defined in Equations 10–12, with flow regime verification given by Equation 13. Alginate (8%) and MeHA (2%) were modelled as generalized Newtonian fluids using the power-law (Ostwald–de Waele) relation,

$$\mu \left(\dot{\gamma }\right)=K{\dot{\gamma }}^{\;n-1},$$ (10)

where $\dot{\gamma }=\sqrt{2\;D:D}$ is the scalar shear rate, K is the consistency index (Pas^n^), and n<1 defines the degree of shear-thinning. As $\dot{\gamma }$ increases near walls and junctions, the apparent viscosity decreases, locally increasing velocity gradients and concentrating wall shear stress.

The rheological parameters (K,n) assigned to alginate and MeHA were selected from published rheological characterisations of comparable printable formulations reported over extrusion-relevant shear-rate ranges (Paxton et al., 2017; Jia et al., 2014; Petta et al., 2020; Highley et al., 2015; Schwab et al., 2020; Rezende et al., 2009; Li et al., 2016; Fu et al., 2011; Chernos et al., 2017; García-Abuín et al., 2011). These values were then applied unchanged in all simulations to enable controlled comparison between nozzle geometries under fixed material models.

To prevent numerical divergence as $\dot{\gamma }\to 0$ , viscosity was bounded as

$$\mu_{\text{reg}}\left(\dot{\gamma }\right)=\min\;\left(\mu_{\max},\;\max\;\left(\mu_{\min},\;K\;{\dot{\gamma }}^{\;n-1}\right)\right),$$ (11)

with $\mu_{\min}=0.001\text{ Pas}$ and $\mu_{\max}=10\text{ Pas}$ . The lower bound avoids unrealistically small viscosities in high-shear zones, while the upper bound limits growth in near-stagnant regions without materially affecting the operative extrusion shear-rate range.

#### 2.4.3 Near-Newtonian bioink (GelMA)

GelMA (10%) was treated as Newtonian over the simulated shear-rate window,

$$\mu \left(\dot{\gamma }\right)\equiv \mu_{\text{G}},$$ (12)

with $\mu_{\text{G}}=0.46\text{ Pas}$ , chosen as a representative apparent viscosity within the range reported for GelMA bioinks at physiological printing temperature (Mendoza-Cerezo et al., 2024; Yue et al., 2015; Billiet et al., 2012; Rastin et al., 2020). Under these conditions, viscosity does not vary appreciably with shear rate, allowing geometry-induced flow redistribution to be isolated without rheology–shear coupling effects.

#### 2.4.4 Laminar-flow verification

Reynolds numbers were computed as

$$Re=\frac{\rho UD}{\mu^{*}},$$ (13)

where $\mu^{*}=\mu_{\text{reg}}\left(\bar{\dot{\gamma }}\right)$ for shear-thinning inks and $\mu^{*}=\mu_{\text{G}}$ for GelMA. Across all geometries and inlet pressures, Re=O(1) or lower. Inertia therefore remains negligible relative to viscous stresses, justifying the steady laminar formulation.

### 2.5 Performance metrics

Performance was assessed using the same four metrics defined in §2.2: (i) wall shear measures (mean and peak WSS) as a proxy for cell safety, (ii) per-outlet and total volumetric flow for throughput and outlet balance, (iii) pressure field and overall pressure drop for hydraulic cost and printer constraints, and (iv) outlet velocity as a complementary indicator of deposition behaviour. Metrics are reported and interpreted using those prior definitions without redefinition, and are discussed separately rather than collapsed into a single objective to reflect the inherent trade-offs between throughput, balance, and shear exposure.

*Wall shear:* Mean WSS ${\bar{\tau }}_{w}$ (area-averaged over the split-block walls) and peak WSS $\tau_{w,\max}$ were used to capture typical shear exposure and local hotspots at junctions and turns. Wall shear stress was evaluated directly from the CFD solution and is used as a comparative indicator of mechanical loading relevant to cell-laden extrusion, consistent with prior studies on shear-mediated cell damage (McCauley et al., 2025; Lemarié et al., 2021; Ramesh et al., 2021).

*Flow rate and balance:* Outlet flow rates $Q_{i}$ quantify throughput and provide the basis for the flow uniformity and imbalance measures in §2.2. Reporting both $\sum_{i}Q_{i}$ and the outlet-to-outlet distribution isolates how geometry affects scalability and deposition uniformity in multi-outlet extrusion.

*Pressure:* Pressure drop Δp between inlet and outlet planes was used as the primary energetic constraint, together with hydraulic power $P_{h}=\Delta p\sum_{i}Q_{i}$ . Pressure contours were used only to localise dominant loss regions; local gauge extrema are shown for interpretation and are not treated as an outlet “suction” metric.

*Velocity:* Velocity fields and outlet-mean velocities (derived from $Q_{i}$ ) were reported as complementary indicators of flow redistribution and potential deposition differences across outlets. For shear-thinning inks, velocity variations are coupled to local viscosity through $\dot{\gamma }$ -dependent rheology, linking outlet balance and shear hotspots to the same underlying flow features.

### 2.6 CFD simulations

Computational fluid dynamics (CFD) is widely used to resolve in-nozzle pressure, velocity, and wall shear stress fields in extrusion bioprinting, which are difficult to access experimentally due to the small scale and opacity of nozzle channels (Abdulwahab et al., 2020; Jahanbakhsh et al., 2020; Schwab et al., 2020; Chand et al., 2022; Xu et al., 2022; Gharraei et al., 2024; Lamberger et al., 2024). Here, CFD provides a controlled basis for comparing multi-outlet nozzle geometries under identical operating conditions.

All simulations were performed in ANSYS Fluent 2025 R1 assuming steady, incompressible, laminar flow. Shear-thinning bioinks were represented using a power-law generalized Newtonian viscosity, while GelMA was treated using a constant-viscosity approximation (Ansys and Inc., 2025). The governing equations are.

∇⋅u=0, (14)

$$\rho \left(u\cdot \nabla \right)u=-\nabla p+\nabla \cdot \tau ,\;\tau =2\;\mu \left(\dot{\gamma }\right)\;D,$$ (15)

where u is the velocity, p the pressure, ρ the density, $D=\frac{1}{2}\left(\nabla u+\nabla u^{⊤}\right)$ the rate-of-strain tensor, and $\dot{\gamma }=\sqrt{2\;D:D}$ the scalar shear rate.

Across all cases, Reynolds numbers based on outlet diameter D , mean outlet velocity U , and an effective viscosity $\mu^{*}$ were O(1) or lower, supporting the laminar assumption. A pressure inlet was applied with $p_{\text{in}}\in \left\{65\text{ mbar},85\text{ mbar},105\text{ mbar}\right\}$ (gauge) and all outlets were set to $p_{\text{out}}=0\text{ mbar}$ (gauge); walls were no-slip, and temperature and gravity effects were neglected.

Each geometry (Y-split and 90° split) and outlet count (N=2,4) was meshed separately with local refinement at junctions and outlets and near-wall layers to resolve velocity gradients. A pressure-based solver with SIMPLE coupling was used. Gradients were computed by least-squares, and second-order spatial discretisation was applied for pressure and momentum. Apparent viscosity was updated from the local shear rate at each iteration following Fluent’s standard non-Newtonian implementation (Ansys and Inc., 2025). For numerical stability at very low strain rates, viscosity limits were enforced for power-law inks. The upper bound ( $\mu_{\max}=10$ Pa ⋅ s) was reached locally in near-stagnant regions for alginate and MeHA, with verification of the realised viscosity range reported in Supplementary S2.

Convergence was assessed by scaled residuals ( $<10^{-5}$ for continuity and momentum), global mass imbalance (<0.1%) , and stabilisation of monitored area-averaged outlet flow rates. Simulations were advanced to approximately $10^{3}$ iterations to ensure steady fields. For representative cases, grid-refinement studies were performed until changes in area-averaged outlet velocity $\bar{U}$ and mean wall shear stress ${\bar{\tau }}_{w}$ were below 1% and 2% , respectively, indicating mesh-independent results (Vivarelli et al., 2025). Unless stated otherwise, identical boundary conditions, material parameters, and meshing strategy were applied across cases to enable direct geometry-to-geometry comparisons. All pressures are reported as gauge relative to the outlet boundary (0 mbar). Inlet pressures are specified in mbar to reflect printer settings, while pressure fields are reported in pascals as returned by the solver (1 mbar=100 Pa) .

## 3 Results

### 3.1 Spatial field analysis

**FIGURE 4:**
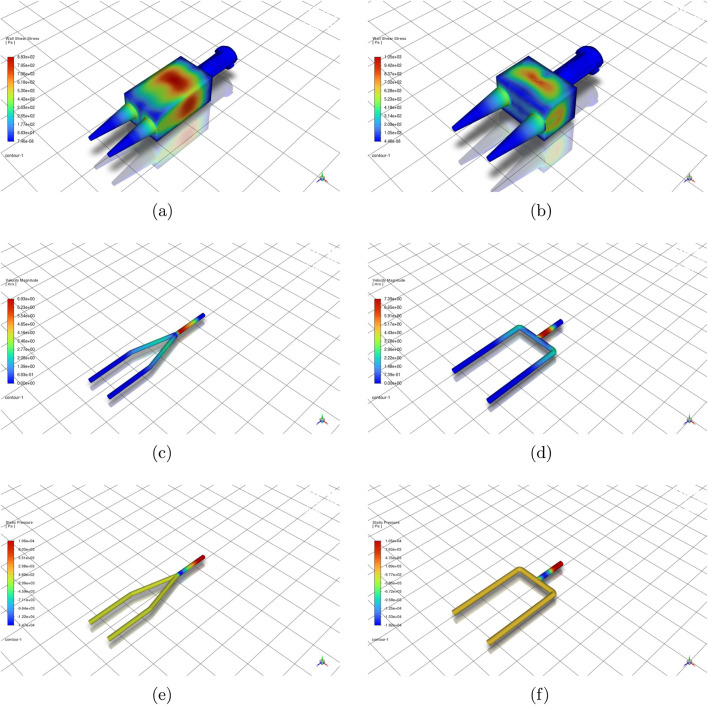
Comparison of 2-outlet nozzle designs (Y-split vs. 90 ° ) across key spatial metrics for Alginate 8% at 105 mbar. **(a)** Wall shear stress (2-outlet, Y-split, Alginate 8%, 105 mbar). **(b)** Wall shear stress (2-outlet, 90 ° , Alginate 8%, 105 mbar). **(c)** Velocity field (2-outlet, Y-split, Alginate 8%, 105 mbar). **(d)** Velocity field (2-outlet, 90 ° , Alginate 8%, 105 mbar). **(e)** Static pressure (2-outlet, Y-split, Alginate 8%, 105 mbar). **(f)** Static pressure (2-outlet, 90 ° , Alginate 8%, 105 mbar).

Figure 4 contrasts the 2-outlet geometries for 8% alginate at 105 mbar. Relative to the 90 ° split, the Y-split shows higher WSS and a more centralised high-velocity core, indicating a larger region of elevated shear within the splitter while sustaining higher branch velocities. Static pressure contours are reported as gauge relative to the outlet boundary $\left(p_{\text{out}}=0\right)$ and are included for qualitative interpretation only. Locally negative gauge values may therefore appear and are not used as a suction metric.

**FIGURE 5:**
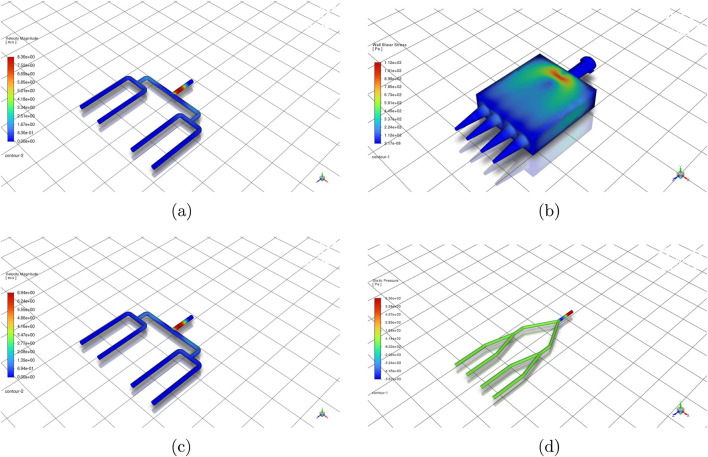
Spatial distribution of velocity, wall shear stress, and static pressure across selected nozzle designs and test cases. **(a)** Velocity field (4-outlet, 90 ° , Alginate 8%, 105 mbar). **(b)** Wall shear stress (4-outlet, Y-split, Alginate 8%, 105 mbar). **(c)** Velocity field (4-outlet, 90 ° , Alginate 8%, 85 mbar). **(d)** Static pressure (4-outlet, Y-split, Alginate 8%, 85 mbar).

Figure 5 presents representative spatial fields for selected four-outlet cases. At 105 mbar, the four-outlet 90 ° design exhibits a visually symmetric velocity field, whereas the four-outlet Y-split shows peak WSS localised at the primary junction, indicating that the dominant shear hotspot persists with increasing outlet count. At lower inlet pressure, the branched network becomes more susceptible to maldistribution, consistent with the outlet-imbalance trends (Figure 7).

Table 3 summarises the performance trends across all simulated nozzle configurations. The 2-outlet 90 ° split achieved the highest flow symmetry, with $I_{Q}$ below 1% for shear-thinning bioinks (alginate and MeHA). GelMA showed greater imbalance due to its higher effective viscosity, which amplifies minor geometric asymmetries. This configuration also produced the lowest wall shear stress (WSS), indicating reduced mechanical loading on encapsulated cells and supporting its suitability for cell-laden bioinks (Shakur et al., 2025).

**TABLE 3:** Qualitative summary of performance trade-offs across nozzle configurations and metrics. Ratings reflect relative behavior from CFD results. Flow balance applies only to multi-outlet designs; the single-outlet control is marked as not applicable.

Configuration	Flow balance	WSS (cell safety)	Pressure loss localisation	Velocity (per outlet)	Efficiency $\left(\eta ={\bar{U}}_{\text{out}}/\Delta p\right)$
Single outlet (control)	–	✓✓	✓✓	✓✓✓	✓
2-Outlet, 90 ° split	✓✓✓	✓✓✓	✓✓✓	✓✓	✓✓✓
2-Outlet, Y-Split	✓✓	✓	✓✓	✓✓	✓
4-Outlet, 90 ° split	✗	✓✓	✓✓	✓	✓✓
4-Outlet, Y-Split	✗	✗	✓	✓	✗

The qualitative ratings in Table 3 indicate relative performance, where more check marks denote better performance within a given metric, and a cross (×) denotes poor performance.

The single-outlet control exhibited the highest outlet velocity per nozzle, as expected from mass conservation under identical pressure boundary conditions. However, its pressure-normalised throughput $\left(\eta ={\bar{U}}_{\text{out}}/\Delta p\right)$ remained lower than that of the 2-outlet configurations. This occurs because the entire volumetric flow passes through a single restrictive channel, increasing downstream viscous losses and total pressure drop relative to delivered outlet velocity.

The 2-outlet Y-split generated higher per-branch velocity than the 4-outlet geometries and supported greater material throughput. However, sharper directional changes at the bifurcation increased local shear stress and produced moderate flow imbalance. These effects arise from asymmetric pressure redistribution at the junction, which concentrates velocity gradients near the inner walls.

Increasing the outlet count to four reduced flow symmetry in both branching strategies. The 4-outlet Y-split produced the highest shear stress concentrations, frequently exceeding 150 Pa, due to compounded bifurcation losses and non-uniform branch resistance. The 4-outlet 90 ° design distributed pressure more evenly and reduced peak WSS relative to the Y-split variant, but its overall performance remained inferior to the 2-outlet configurations.

These results support a task-oriented nozzle selection strategy. The 2-outlet 90 ° split provides optimal balance between flow symmetry, low shear stress, and pressure-normalised throughput, making it suitable for precision and cell-sensitive applications. The 2-outlet Y-split favours higher deposition rates where moderate shear increases are acceptable. Four-outlet geometries introduce elevated shear gradients and reduced symmetry, limiting their use for delicate bioinks without active flow control. All CAD geometries, CFD models, and analysis scripts are available at https://github.com/Ceurda/bioprinter-nozzle-paper.

### 3.2 Cross-condition comparison across bioinks and inlet pressures

**FIGURE 6:**
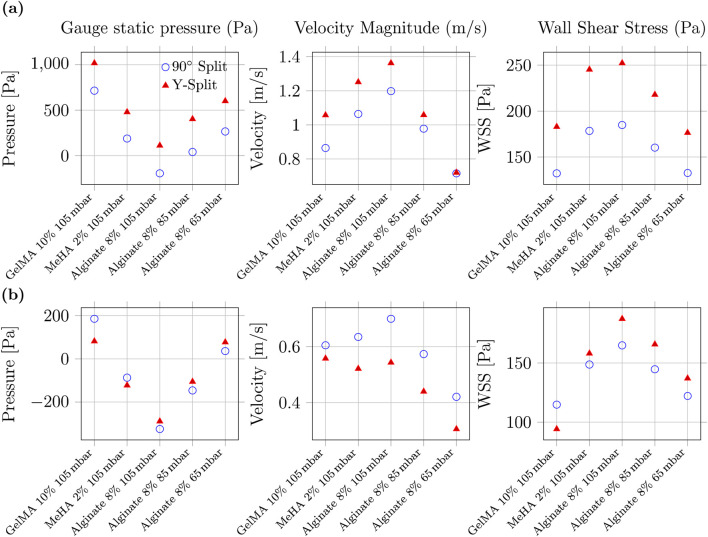
CFD-based performance comparison of Y-split and 90 ° nozzles. **(a)** Two-outlet configurations. **(b)** Four-outlet configurations. Columns show static pressure, velocity magnitude, and wall shear stress. Static pressure is reported as gauge relative to the outlet boundary $\left(p_{\text{out}}=0\right)$ and is included to visualise loss localisation rather than absolute pressure magnitude.

In Figure 6a, static pressure contours indicate that pressure losses in Y-split geometries are primarily localised at the primary bifurcation, whereas in the 90 ° designs losses are distributed across successive bends and straight sections. These differences reflect local acceleration, turning, and junction effects within the nozzle and are used here solely to diagnose loss localisation rather than absolute pressure magnitude. Quantitative comparison between designs is therefore based on Δp , as defined in §2.2.

In terms of velocity, Y-split designs also produce consistently higher outlet speeds, particularly for lower-viscosity MeHA and alginate. At 105 mbar, Y-split nozzles exceed 1.3 m/s, compared with approximately 1.2 m/s in 90 ° designs. This difference reflects reduced minor losses associated with smoother bifurcation in the Y-split geometry.

However, these benefits come at a cost: wall shear stress (WSS) is consistently higher in the Y-split configuration, particularly under high inlet pressure and low-viscosity conditions. Maximum WSS reaches approximately 250 Pa for 8% alginate at 105 mbar. Reported shear–viability thresholds in extrusion bioprinting vary widely with cell type, bioink formulation, and nozzle residence time. Accordingly, WSS is interpreted here as a comparative indicator of relative shear exposure rather than as a universal damage threshold (Elango and Zamora-Ledezma, 2025). In this comparative context, the 90 ° nozzle exhibits a more conservative shear profile across all tested cases, supporting its suitability for cell-laden bioinks.

Figure 6b extends the analysis to four-outlet configurations. In four-outlet configurations, both geometries exhibit reduced outlet velocity and increased sensitivity to pressure and bioink rheology. The static-pressure fields become more spatially uniform with increasing outlet count, indicating redistribution of hydraulic losses across additional branches. Yet, the Y-split design continues to exhibit slightly higher outlet velocities, mirroring trends from the two-outlet case.

The velocity advantage of the Y-split design diminishes in the four-outlet configuration. The performance gap between geometries narrows, particularly under lower-pressure conditions (e.g., 65 mbar with 8% Alginate), where both designs converge to similar velocity magnitudes (0.5–0.6 m/s). This trend suggests that increasing outlet count reduces the benefit of smoother Y-transitions, likely due to cumulative minor losses and symmetry degradation.

The WSS trends, however, persist: Y-split geometries still produce higher peak shear values, especially for high-pressure, low-viscosity inputs. The 90 ° nozzle again demonstrates a more conservative shear profile, implying safer use for bioinks with embedded living cells. For example, GelMA at 105 mbar yields approximately 110 Pa in WSS for the 90 ° layout versus 135 Pa in the Y-split.

### 3.3 Impact of different materials

**FIGURE 7:**
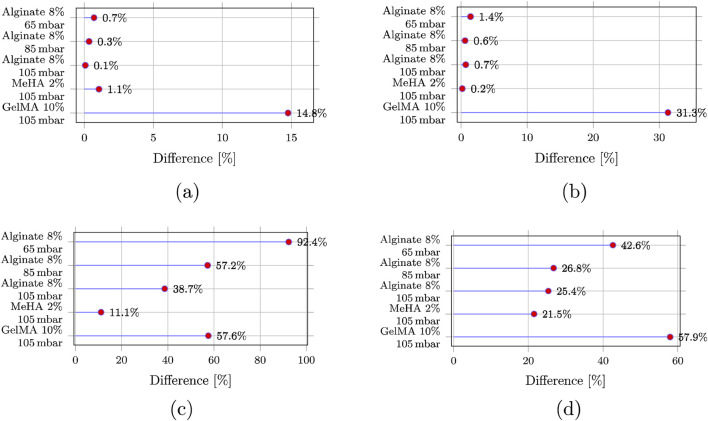
Outlet flow imbalance was quantified using the flow-rate imbalance metric $I_{Q}$ defined in §2.2, computed from the volumetric flow rates $Q_{i}$ on each outlet plane. Because all outlet areas are identical, the same trends are reflected in outlet mean velocities, but all imbalance values reported here are based on $Q_{i}$ for consistency with the primary throughput metric. Figure 7 reports the flow-rate imbalance metric $I_{Q}$ across all outlet counts and operating cases. Subfigures **(a)** and **(b)** compare the 2-outlet configurations, while **(c)** and **(d)** extend the comparison to 4-outlet designs. **(a)** 90 ° split, 2 outlets. **(b)** Y-split, 2 outlets. **(c)** 90 ° split, 4 outlets. **(d)** Y-split, 4 outlets.

Figure 7 quantifies outlet non-uniformity using the imbalance metric $I_{Q}$ across bioinks and inlet pressures for the two- and four-outlet designs. For the two-outlet geometries (Figures 7a,b), $I_{Q}$ remains negligible for the shear-thinning bioinks (alginate and MeHA), staying below 1.5% across all tested inlet pressures. GelMA is the clear outlier: at 105 mbar, $I_{Q}$ increases to 14.8% for the 90 ° split (Figure 7a) and 31.3% for the Y-split (Figure 7b), indicating substantially higher sensitivity of the split to the GelMA operating regime.

In contrast, the four-outlet configurations (Figures 7c,d) exhibit a pronounced loss of uniformity across all materials. Flow-rate imbalance exceeds 20% in all four-outlet cases and reaches 57.9% for GelMA in the four-outlet Y-split configuration (Figure 7d). The four-outlet 90 ° manifold is additionally pressure-sensitive, with asymmetry worsening at lower inlet pressure and peaking at 92.4% for alginate at 65 mbar (Figure 7c). Overall, Figure 7 shows that two-outlet designs provide robust outlet uniformity for shear-thinning bioinks, whereas four-outlet splitting introduces strong sensitivity to rheology and inlet pressure that would likely require active compensation to maintain uniform deposition.

### 3.4 Integrated performance metrics and trade-offs

**FIGURE 8:**
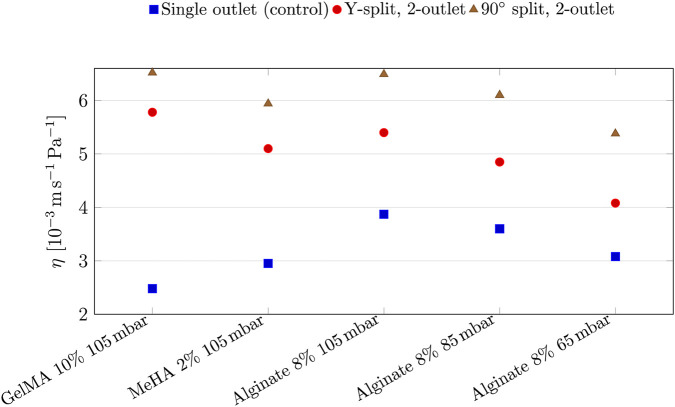
Efficiency index $\eta ={\bar{U}}_{\text{out}}/\Delta p$ for single-outlet (control) and 2-outlet split designs across bioinks and inlet pressures.

Figure 8 compares pressure-normalised throughput η across all tested conditions for two-outlet nozzles. Across all cases, the 90 ° design exhibits higher efficiency values than the Y-split counterpart. This indicates that although the Y-split achieves higher outlet velocities, it does so with a greater pressure cost (higher Δp ), reducing pressure-normalised throughput under fixed pneumatic actuation. The 90 ° design maintains more favourable pressure-normalised throughput, indicating lower hydraulic cost under fixed pneumatic actuation. Cell viability considerations are assessed separately using mean and peak wall shear stress (Suwannaphan et al., 2025).

**FIGURE 9:**
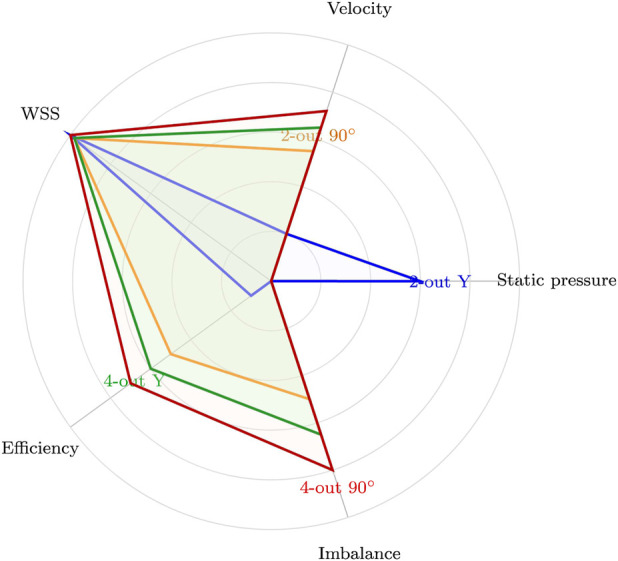
Radar plot comparing normalized values across five performance dimensions for 2- and 4-outlet Y-split and 90 ° configurations using Alginate 8% at 105 mbar.

Figure 9 presents a radar chart summarising five performance metrics, velocity, wall shear stress (WSS), efficiency, and outlet imbalance—for Alginate 8% at 105 mbar. The 90 ° two-outlet configuration shows a balanced profile across all axes, indicating consistent performance with low shear and minimal imbalance. In contrast, the four-outlet Y-split achieves higher throughput (higher outlet velocity) but exhibits reduced pressure-normalised throughput and elevated shear, which may adversely affect cell viability.

These multidimensional comparisons reinforce the need for task-specific nozzle selection rather than a single globally optimal design. For high-precision or viability-sensitive applications, minimising shear stress and outlet imbalance is the primary design priority. For rapid deposition or acellular support materials, higher velocity and pressure may be prioritised, justifying the use of geometries such as Y-splits despite their shear cost.

## 4 Discussion

Extrusion bioprinting performance is difficult to measure in narrow, opaque nozzles, as optical velocimetry (PIV/µPIV) faces access and uncertainty limitations, particularly for scattering, cell-laden bioinks. CFD is therefore routinely used to resolve pressure, wall shear stress (WSS), and velocity fields and to compare candidate designs under controlled conditions (Abdulwahab et al., 2020; Chand et al., 2022). The present simulations show that nozzle geometry strongly influences flow dynamics, stress distribution, and extrusion efficiency across biomaterials and inlet pressures. Two-outlet nozzles exhibit superior flow symmetry, lower WSS, and higher efficiency than four-outlet counterparts under comparable operating conditions. Geometry and outlet size jointly govern WSS, pressure drop, and flow rate, which directly influence viability and print fidelity in extrusion bioprinting (Chand et al., 2022; Schwab et al., 2020).

The 90 ° 2-outlet design provides the most balanced performance. As shown in Figures 4, 6, it maintains low outlet imbalance $\left(I_{Q}<1\%\right)$ , moderate internal velocities, and the lowest WSS across all tested bioinks. These characteristics are favourable for cell-laden systems, where limiting mechanical loading is important for preserving viability (Wei et al., 2025; Lombardi et al., 2025; Li et al., 2025; Ahmad et al., 2025). Mechanistically, cell survival decreases with increasing shear magnitude and cumulative exposure time; nozzle geometry, pressure, extrusion rate, and viscosity collectively determine this mechanical burden (Xu et al., 2022). Designs that limit sustained high-shear regions at matched flow targets therefore better preserve viability (Chand et al., 2022).

Wall shear stress contours correspond to the internal fluid–solid interface of the nozzle channels. The rendered surfaces represent the CFD boundary of the fluid domain; no shear values were evaluated on external structural walls.

Wall shear stress represents the maximum local mechanical loading within laminar pressure-driven flow. In cylindrical channels, shear magnitude decreases monotonically from the wall toward the centreline, and cells transported within the core experience lower shear than the reported wall values. The WSS values presented here should therefore be interpreted as conservative upper-bound indicators of mechanical exposure rather than spatially averaged cell stress. Incorporating cross-sectional or residence-time-weighted shear metrics would provide additional biological resolution in future work.

The efficiency index (Figure 8) further supports the 90 ° 2-outlet configuration by demonstrating superior pressure-normalised throughput at comparable operating pressures. In contrast, the Y-split 2-outlet design produces higher outlet velocities, which may be advantageous for rapid deposition or acellular constructs. However, this occurs alongside elevated WSS and reduced flow balance at higher viscosities and pressures (Figure 7). The 31% imbalance observed during GelMA extrusion illustrates how asymmetric flow can compromise deposition uniformity, consistent with printability frameworks linking rheology–process mismatch to filament instability and pore distortion (Schwab et al., 2020).

Increasing the outlet number to four introduces hydraulic penalties. Both Y-split and 90 ° four-outlet geometries exhibit reduced velocities and substantial outlet imbalance (often exceeding 50%), particularly under low-pressure alginate conditions. These trends indicate accumulated minor losses and symmetry disruption within multi-branch junctions. Although the 4-outlet 90 ° configuration slightly improves uniformity relative to its Y-split counterpart, neither matches the stability of two-outlet designs. The radar plot in Figure 9 consolidates these trade-offs, demonstrating that no single geometry dominates across all performance metrics. Design selection must therefore reflect the biological tolerance and throughput requirements of the intended application.

Recent studies emphasise application-specific pairing of rheology, nozzle geometry, pressure, and speed to achieve fidelity and viability targets (Schwab et al., 2020). The present results support a task-dependent nozzle selection strategy in which geometry is chosen based on rheological constraints and biological tolerance thresholds (Chirianni et al., 2024; Hidaka et al., 2024). Designs prioritising minimal WSS and high symmetry are preferable for cell-laden constructs, whereas higher-throughput configurations may be acceptable for acellular deposition.

Although internal channel velocities may exceed external stage translation speeds during printing, these quantities are not directly comparable. The reported values correspond to local internal fluid velocities within the nozzle, which scale with volumetric flow rate divided by channel cross-sectional area and are therefore expected to differ from external deposition speeds. The applied pressure range (65 mbar–105 mbar) lies within pneumatic extrusion regimes used for hydrogel-based printing systems. The simulations were pressure-controlled to enable consistent geometry comparison under identical loading conditions. The results are therefore intended for relative performance assessment rather than replication of a specific printer translation speed.

All simulations assumed steady, laminar flow under constant inlet pressure. This is appropriate for low-Reynolds-number hydrogel extrusion but does not capture transient effects such as pressure ramp-up, regulator fluctuations, or start–stop toolpaths that may transiently increase shear exposure or imbalance. The study remains computational and lacks experimental validation. Fabricating the two- and four-outlet designs and performing controlled extrusion tests would directly assess predicted flow balance and pressure–flow relationships. Geometric dimensions were fixed to one representative printer configuration, and only two- and four-outlet layouts were evaluated. Extending the same modelling framework to higher outlet counts would determine when passive geometric symmetry becomes insufficient. The present study evaluates nozzle performance from a fluid-mechanical perspective. Biological outcomes are inferred indirectly through mechanical indicators such as wall shear stress; direct validation under cell-laden printing conditions remains a necessary next step. While experimental validation is required to confirm absolute magnitudes, the qualitative ranking between geometries is expected to remain stable due to the symmetry-driven nature of the observed flow splitting.

## 5 Conclusion

Task-specific multi-outlet nozzle designs were quantified using CFD simulations to quantify how multi–outlet nozzle geometry affects flow uniformity, WSS, and extrusion efficiency across representative bioinks and inlet pressures. The results show that nozzle geometry is a dominant factor governing extrusion performance. The two-outlet 90 ° nozzle provides the best balance of flow symmetry, low WSS, and efficiency, indicating suitability for cell-laden hydrogel printing. The two-outlet Y-split achieves higher outlet velocities and throughput but incurs elevated shear, making it more appropriate for acellular or support materials. Increasing to four outlets consistently degraded balance and efficiency, highlighting the limits of passive scaling. Overall, these findings support our task-specific framework for selecting nozzle geometry based on biological and process constraints.

## Data Availability

The datasets presented in this study can be found in online repositories. The names of the repository/repositories and accession number(s) can be found below: https://github.com/Ceurda/bioprinter-nozzle-paper.
