# Supplementary material for: Task-specific computational fluid dynamics evaluation of multi-outlet extrusion nozzles for bioprinting
Source: Front Bioeng Biotechnol. 2026 Apr 17;14:1784513. doi: 10.3389/fbioe.2026.1784513 (PMC13133019; doi:10.3389/fbioe.2026.1784513)
Supplement: Supplementary file 1 [file DataSheet1.pdf]

# Supplementary material

### Supplementary File S1: Spatial field panels.

Table 4: Sensitivity of effective viscosity $\mu_{\text{eff}}$ evaluated at a representative shear rate of $\dot{\gamma} = 1000\ \text{s}^{-1}$. A $\pm 20\%$ variation in the consistency index $K$ and a $\pm 0.05$ variation in the flow behaviour index $n$ were tested for power-law bioinks. GelMA was modeled as Newtonian and therefore remains constant.

| **Bioink (model)** | **Baseline $\mu_{\text{eff}}$ [Pa·s]** | $K$ **−20%** | $K$ **+20%** | $n \pm 0.05$ |
|---|---|---|---|---|
| Alginate 8% (Power-law: $K = 56.9$, $n = 0.322$) | 0.526 | 0.421 (−20%) | 0.631 (+20%) | 0.372 (−29%) \| 0.743 (+41%) |
| MeHA 2% (Power-law: $K = 12.4$, $n = 0.55$) | 0.554 | 0.443 (−20%) | 0.665 (+20%) | 0.392 (−29%) \| 0.782 (+41%) |
| GelMA 10% (Newtonian: $\mu = 0.46$) | 0.460 | – | – | – |

Table 5: Geometry specification: 2-outlet 90° split nozzle (splitting block only; all dimensions in mm).

| Parameter | Value |
|---|---|
| Inlet diameter $D_{\text{in}}$ | 1.35 |
| Inlet straight length $L_{\text{in}}$ | 8.50 |
| Channel diameter $D_{\text{b}}$ | 1.35 |
| Straight run from junction to bend start $L_{\text{pre}}$ | 7.745 |
| Bend angle | 90° |
| Bend radius (centreline) $R_c$ | 1.00 |
| Straight run from bend end to outlet plane $L_{\text{post}}$ | 5.355 |
| Outlet diameter at split-block exit $D_{\text{out}}$ | 1.35 |

Table 6: Geometry specification: 2-outlet Y-split nozzle (splitting block only; all dimensions in mm).

| Parameter | Value |
|---|---|
| Inlet diameter $D_{\text{in}}$ | 1.35 |
| Inlet straight length $L_{\text{in}}$ | 8.50 |
| Channel diameter $D_{\text{b}}$ | 1.35 |
| Y-split bifurcation angle $\theta_Y$ | 30° |
| Branch angle relative to centreline | 15° |
| Branch centreline length (split to outlet plane) $L_{\text{branch}}$ | 15.611 |
| Branch diameter $D_{\text{branch}}$ | 1.35 |
| Outlet diameter at split-block exit $D_{\text{out}}$ | 1.35 |

Table 7: Geometry specification: 4-outlet 90° split nozzle (splitting block only; all dimensions in mm).

| Parameter | Value |
|---|---|
| Inlet diameter $D_{\text{in}}$ | 1.35 |
| Inlet straight length $L_{\text{in}}$ | 8.50 |
| Parent channel diameter $D_{\text{b1}}$ | 1.35 |
| *First split (parent → 2 branches)* | |
| Straight run from first split centre to bend start $L_{\text{pre},1}$ | 11.343 |
| Bend angle (tier 1) | 90° |
| Bend radius (centreline) $R_{c,1}$ | 1.00 |
| Bend centreline arc length (tier 1) $L_{\text{arc},1} = \frac{\pi}{2} R_{c,1}$ | 1.571 |
| Inter-tier straight length $L_{\text{inter}}$ | 3.00 |
| *Second split (each branch → 2 outlets)* | |
| Straight run from second split centre to bend start $L_{\text{pre},2}$ | 4.35 |
| Bend angle (tier 2) | 90° |
| Bend radius (centreline) $R_{c,2}$ | 1.00 |
| Bend centreline arc length (tier 2) $L_{\text{arc},2} = \frac{\pi}{2} R_{c,2}$ | 1.571 |
| Outlet diameter at split-block exit plane $D_{\text{out,SB}}$ | 1.35 |
| Outlet straight length inside split block $L_{\text{out,SB}}$ | 0.000 |
| Outlet plane used for reporting | Needle outlet plane (Table 9) |

Table 8: Geometry specification: 4-outlet Y-split nozzle (splitting block only; all dimensions in mm).

| Parameter | Value |
|---|---|
| Inlet diameter $D_{\text{in}}$ | 1.35 |
| Inlet straight length $L_{\text{in}}$ | 8.50 |
| Parent channel diameter $D_{\text{b1}}$ | 1.35 |
| *First split (parent → 2 branches)* | |
| Y-split bifurcation angle $\theta_{Y,1}$ | 60° |
| Branch angle relative to parent centreline | 30° |
| Centreline length (first split centre to next straight) $L_1$ | 15.015 |
| Branch diameter (tier 1) $D_{\text{b2}}$ | 1.35 |
| Inter-tier straight length $L_{\text{inter}}$ | 7.071 |
| *Second split (each branch → 2 outlets)* | |
| Y-split bifurcation angle $\theta_{Y,2}$ | 30° |
| Sub-branch angle relative to parent | 15° |
| Centreline length (second split centre to split-block exit plane) $L_2$ | 13.928 |
| Outlet diameter at split-block exit plane $D_{\text{out,SB}}$ | 1.35 |
| Outlet straight length inside split block $L_{\text{out,SB}}$ | 0.000 |
| Outlet plane used for reporting | Needle outlet plane (Table 9) |
| Outlet taper angle $\alpha$ | 0 (no taper) |
| Taper length $L_\alpha$ | – |

Table 9: Shared needle geometry used for all outlet channels (all dimensions in mm).

| Parameter | Value |
|---|---|
| Needle internal diameter at inlet $D_{\text{needle,in}}$ | 1.35 |
| Needle internal diameter at outlet $D_{\text{needle,out}}$ | 1.35 |
| Needle centreline length $L_{\text{needle}}$ | 14.60 |
| Internal taper | None (constant internal diameter) |
| Taper length $L_{\alpha,\text{needle}}$ | – |
| Taper half-angle $\alpha_{\text{needle}}$ | – |
| Outlet plane used for reporting | Needle exit plane |

# Supplementary S2: Rheological parameter selection and viscosity bounds verification

## Rheological parameter selection

Rheological parameters were selected from experimentally reported characterisations of printable hydrogel formulations in the literature. These studies report shear-thinning behaviour for alginate and hyaluronic-acid–based bioinks across extrusion-relevant shear-rate ranges, typically $\sim 10^1$–$10^4$ s$^{-1}$. Consistent with these measurements, alginate (8%) and MeHA (2%) were represented as generalised Newtonian fluids using the power-law (Ostwald–de Waele) relation

$$\mu(\dot{\gamma}) = K\dot{\gamma}^{n-1}.$$

The parameters $(K, n)$ were selected within ranges reported for comparable formulations in published rheological studies of alginate and hyaluronic-acid-based hydrogels. These values reproduce the experimentally observed shear-thinning behaviour of extrusion bioinks and are commonly used in computational modelling of bioprinting flows.

For 10% GelMA, which exhibits comparatively weak shear dependence near physiological printing temperatures, a constant viscosity $\mu_G = 0.46\,\text{Pa s}$ was adopted based on steady-shear measurements reported for GelMA bioinks at 37°C.

The selected parameters were applied consistently across all CFD simulations. The objective of the rheological modelling in this study is comparative evaluation of nozzle geometries under representative bioink behaviour rather than calibration of a specific material formulation.

## Shear-rate relevance

CFD-resolved shear rates within the split-block were primarily $\mathcal{O}(10^2$–$10^4)$ s$^{-1}$, depending on inlet pressure and nozzle layout. This range is consistent with extrusion-reported shear rates for comparable hydrogel systems in the literature and therefore falls within the regime over which the adopted power-law parameters are typically reported.

## Viscosity bounds verification

Power-law models predict unbounded viscosity as $\dot{\gamma} \to 0$. To ensure numerical stability and prevent unphysical viscosity growth in near-stagnant regions, bounded viscosity limits were enforced in the solver.

To confirm that these limits did not distort the solution, the minimum and maximum strain-rate magnitudes encountered in converged simulations were extracted, and the corresponding effective viscosity range was evaluated using the adopted constitutive relations.

Table 10: Realised strain-rate ranges across all simulated geometries and inlet pressures, indicating when imposed viscosity bounds were activated in the solver.

| Bioink | $\dot{\gamma}_{\min}$ [s$^{-1}$] | $\dot{\gamma}_{\max}$ [s$^{-1}$] | $\mu_{\text{eff}}$ [Pa·s] | Limit active? |
|---|---|---|---|---|
| 8% Alginate | $9.82 \times 10^{-3}$ | $2.2 \times 10^{5}$ | 0.013–10 | Upper |
| 2% MeHA | $9.98 \times 10^{-3}$ | $3.17 \times 10^{5}$ | 0.040–10 | Upper |
| 10% GelMA | – | – | 0.46 | No |

In both shear-thinning cases, near-stagnant regions generate very low strain rates, activating the imposed upper viscosity bound. The reported viscosity ranges therefore represent solver-bounded values rather than the unbounded analytical limit of the ideal power-law model.

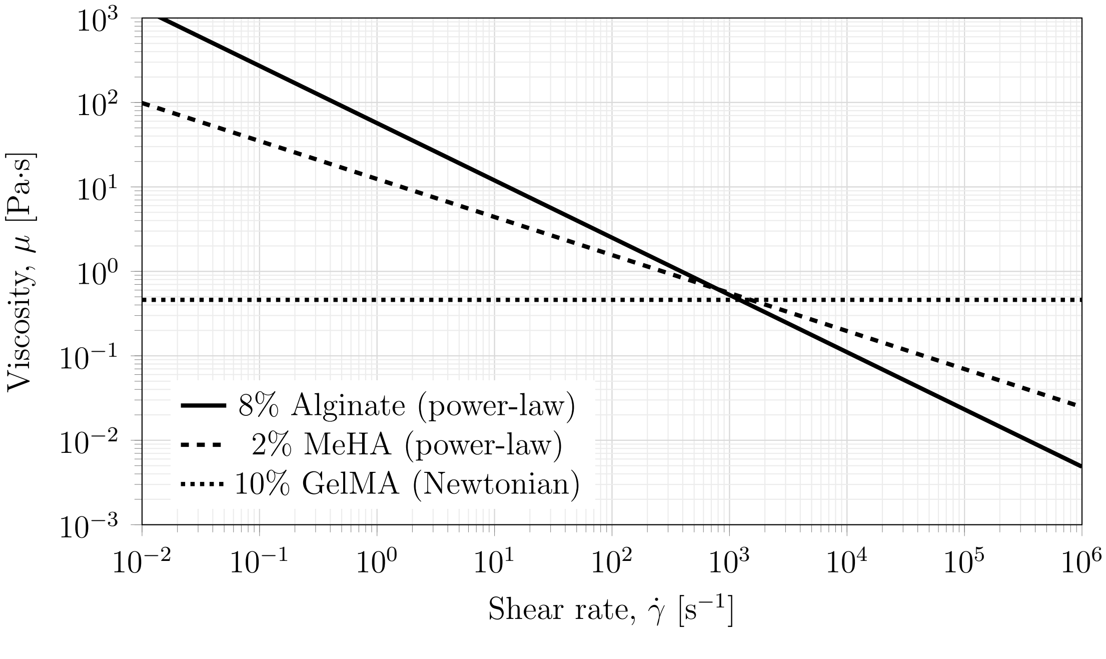


Figure 10: Viscosity–shear-rate relations used in the CFD simulations. Power-law models for 8% alginate and 2% MeHA and the constant viscosity used for 10% GelMA are shown over the shear-rate regime relevant to extrusion-based bioprinting.
